# Sanctions and opportunities: Factors affecting China's high-tech SMEs adoption of artificial intelligence computing leasing business

**DOI:** 10.1016/j.heliyon.2024.e36620

**Published:** 2024-08-22

**Authors:** Wei Sun, Alisher Tohirovich Dedahanov, Wei Ping Li, Ho Young Shin

**Affiliations:** a Management School, Henan University of Urban Construction, Pingdingshan, China; b Business School, Central Asian University, Tashkent, Uzbekistan; c National Cyber Security Experimental Center, Railway Police College, Zhengzhou, China; d School of Business, Yeungnam University, Gyeongsan, South Korea

**Keywords:** Task technology fit, UTAUT, SME, Innovativeness, Artificial intelligence, Computing power leasing

## Abstract

Due to sanctions, more Chinese high-tech SMEs are turning to rent AI computing power through cloud service providers. Therefore, it is necessary to give a variety of suggestions for China's high-tech SMEs to better develop AI applications through computing power leasing. Because traditional theories are difficult to explain this new technology adoption behavior, this research combines and extends TTF and UTAUT2 theories to take an empirical research. A total of 387 questionnaires were received, of which incomplete questionnaires and invalid questionnaires were issued, leaving 281 valid questionnaires. The results indicate that SME innovativeness, perceived risk, performance expectancy, price value and task technology fit are all significantly related to usage, whereas task technology fit moderates the other relationships significantly. Results give a variety of suggestions for China's high-tech SMEs to better develop AI applications through computing power leasing in the context of sanctions. This study not only suggests ways to increase the competitiveness of SMEs by optimizing leasing services but also give directions in investors' investment decisions. The findings are also applicable to the large-scale application of China's domestic AI chips in computing power leasing scenarios in the future.

## Highlights

• Providing ways out for the development of countries and enterprises sanctioned by science and technology.
• Providing a shared business model of artificial intelligence technology development platform.
• A new type of research model is designed by integrating two theoretical models.
• It puts forward a variety of effective suggestions for global high-tech AI SMEs to save operating costs.

## 1 Introduction

Recently, NVIDIA Corporation officially launched the computing power leasing service solution, which is jointly created by NVIDIA Corporation and the world's top cloud service providers such as Microsoft Cloud, Google Cloud, and Oracle, aiming to solve the current situation of imbalance of AI computing power resources. Instead of procuring, deploying, and managing complex on-premises infrastructure, enterprises can use NVIDIA Corporation's AI supercomputing dedicated clusters and supporting software through cloud leasing to help enterprise customers reduce costs and increase efficiency. In addition to NVIDIA Corporation's Cloud platform, Google Cloud AI Platform, Amazon Corporation's Deep Learning system, Microsoft Azure, and IBM Watson Studio all offer similar services that support machine learning training, inference, and more. Computing power leasing is a business model that rents computing power, that is, some enterprises build their own computing power centers by purchasing computing power servers, and then rent computing power to other enterprises to collect rent. Computing power leasing is suitable for artificial intelligence, big data analysis, high-performance computing and other industries, such as unmanned driving, pharmaceutical research and development, intelligent security, urban governance and safety production. Trapped by the United States' increased export ban and technical restrictions on China's semiconductor chip industry, which have led to challenges to scientific autonomy [1], many graphics card manufacturers have been banned from supplying mainstream high-end AI chips required for large model training and inference to China, so China's short-term computing power demand is much higher than computing power supply. Relatively wealthy countries are highly resistant to foreign policy sanctions [2], so China is using its own methods to develop its own AI computing power leasing. For many small and medium-sized enterprises in China, not only do they not have the opportunity to procure the high-end AI chips they need, but they also do not have the financial resources to bear the high operation and maintenance costs of their own data centers, so they could only lease computing power from manufacturers such as computing cloud services. Although economic sanctions may lead to a decline in related industries [3], China's computing power leasing industry is overcoming difficulties and developing on a large scale.

Unlike sweeping sanctions on North Korea, Russia and other groups [[4], [5], [6], [7], [8], [9], [10]], the U.S. Department of Commerce has only imposed stricter restrictions on semiconductor products exported to China. This move has had a huge impact on China's AI industry, especially for intelligent computing companies that provide computing power rental services. The intelligent computing company is a platform that provides cloud-based AI computing services, allowing users to use high-performance AI chips for model training and inference on demand. Due to the ban in the United States, the intelligent computing center is facing the dual pressure of insufficient chip supply and rising costs, and has to increase the price of computing power leasing. For SMEs and institutions that lease computing power, this means they need to pay more to get the computing power they need, which can have an impact on their business costs. The skyrocketing price of computing power leasing may prompt some small and medium-sized enterprises and institutions to reassess their computing needs or try to cope with the increase in rental prices by improving efficiency and reducing costs.

In this context, the contribution is to identify the factors affecting the leasing computing power of SMEs in Chinese mainland, and then analyze the factors and give suggestions. Chinese government is increasing investment in computing infrastructure, building more data centers, and improving the performance of domestically produced AI chips and servers. First, the relevant research theories are reviewed, and then different theories are combined to explain the research objects, and finally quantitative conclusions are drawn based on questionnaires from different industries. The conclusions of this study help governments to make decisions by analyzing the needs of SMEs and thus avoid wasteful investment. By analyzing the factors affecting the acceptance of computing power leasing by SMEs in Chinese mainland, it can help foreign AI chip suppliers and computing power leasing companies better find opportunities to legally circumvent trade sanctions and prepare for participating in the world's largest computing power leasing market in Chinese mainland. By analyzing the factors affecting the acceptance of computing power leasing by SMEs in Chinese mainland, it can help foreign AI chip suppliers and computing power leasing companies better find opportunities to legally circumvent trade sanctions and prepare for participating in the world's largest computing power leasing market in Chinese mainland. Based on the analysis of questionnaire survey data of small and medium-sized enterprises in China, this study provides real data and suggestions on the demand side of computing power leasing for multinational investment companies that are optimistic about the future. Based on these suggestions, mature multinational investment firms can formulate various strategies in advance to gain a competitive advantage in the future competition in the Chinese mainland computing power leasing market.

## 2 Literature review

Artificial intelligence (AI) has been widely used in all walks of life in developed countries, and although China is relatively lagging behind in AI development, AI will bring huge growth opportunities to some of China's key industries, especially in industries such as automotive, securities [11], transportation, finance technology and logistics, manufacturing, healthcare [12] and life sciences, and enterprise software [13]. Application of generative AI means gaining a greater advantage in the fierce market competition for small and medium-sized enterprises. It can help companies automate and intelligently produce and improve production efficiency [14], product quality and other organizational activities [15]. For example, with machine learning, companies can quickly identify market needs and consumer preferences to accurately determine marketing strategies and product directions. At the same time, with these innovative technologies [16] such as natural language processing, image recognition and AI phone applications, businesses can optimize the customer service experience and increase customer satisfaction and loyalty.

### 2.1 SME content analysis

However, China's SMEs also face many challenges when facing the wave of generative AI. First of all, the high technical threshold and investment [17] are the biggest problems. Generative AI requires strong technical capabilities and R&D capabilities [17], which many SMEs lack. Secondly, data security and privacy protection issues are also a major challenge. As more businesses start using generative AI technology, the risk of data breaches and privacy breaches increases. Additionally, the computing power of AI consumes a lot of electricity, which can increase carbon emissions [18,19]. Finally, SMEs need to face the problem of increased market competition. As more and more enterprises pour into the track of intelligent transformation, market competition is becoming more and fiercer, and small and medium-sized enterprises need to continue to innovate and improve their own strength to gain a foothold. Cost reduction and efficiency increase are the most urgent needs of small and medium-sized enterprises in the short term. Promoting industry innovation is the long-term expectation of small and medium-sized enterprises for technology. If the use of AI can lower the threshold for entrepreneurship, it may lead to intensified competition in the industry. Government support is essential [20], and the Chinese government is actively supporting the development of high-tech SMEs, including policy support, tax exemptions, talent training and foreign technology introduction.

Small and medium-sized enterprises have made great contributions to the national tax revenue, and have created considerable gross national product, fixed asset investment and foreign investment, making outstanding contributions to promoting the sustainable development of the national economy. Small and medium-sized enterprises (SMEs) are an important subject of scientific and technological innovation, and they also provide a large number of employment opportunities for the society. Table 1 summarizes some of the previous empirical studies on SMEs, which not only provide recommendations for the development of central enterprises from various aspects, but also demonstrate the importance of SMEs to the overall national economy. Drawing on these research results will help to provide rationality for this study and enrich the research model framework.

**Table 1:** Previous researches about SME.

Authors	Research factors	Research Object
Lopez-Valeiras et al. (2016) [21]	Process innovation, Organizational innovation	Management control systems
Pesamaa, (2017) [15]	Innovativeness, Controls	Future growth
Hameed et al. (2018) [22]	External knowledge, Internal innovation	Open innovation performance
Tadesse & Murthy (2018) [23]	Effectiveness, Credibility, Risk	Investment revisions
Eikelenboom & Jong (2019)	Threat, Capabilities, Leadership	Performances
Aboelmaged & Hashem, (2019) [18]	Capacity, Capital, Collaboration	Green innovation adoption
Bongini (2019) [24]	Tangibility, dependence, profitability	Market-based finance
Lecerf & Omrani (2020) [25]	Innovation, E-CRM	SME Internationalization
Eldridge et al. (2021) [26]	Innovation, Growth opportunity, Financial performance	Use of equity crowd-funding
Kassa & Mirete (2022) [27]	Training, Leadership, Government support	SME Innovation
Ayinaddis (2022) [28]	Risk, Culture, Cost, Size, Human resource	SME Innovation
Handrito et al. (2023) [29]	Achievement, Supportiveness	SME's long-term orientation
Asad et al. (2023) [30]	Innovation, Orientation, Quality management	Performance of SME

### 2.2 Leasing content analysis

Computing power leasing is the leasing of computing power. Some companies spend huge sums of money to buy computing power servers, build their own computing centers, and then rent them to other enterprises on a monthly or annual basis. Many small and medium-sized enterprises want to start AI business, but the cost of self-built computing power is very high, so they choose to lease computing power. There is no difference between computing power leasing and other types of leasing, except that the billing method is more flexible. Lessors usually charge based on the number and length of computed graphics cards leased by the user. A server generally consists of 8 computing graphics cards, and the higher the performance and number of computing graphics cards, the higher the hourly charge. Due to U.S. export sanctions on China's high-performance graphics cards, China's current leasing prices are higher than those in the United States. Table 2 shows a study on a variety of rental products, which helps to deepen the understanding of calculating leases and facilitates the development of research models.

**Table 2:** Previous researches about leasing.

Authors	Leased Property	Research Object
Synek & Koenigstorfer (2018) [31]	Bicycle leasing	Leasing strategy
Liu et al. (2020) [32]	Land lease	Land lease agreement
Huang et al. (2021) [33]	Electric vehicles	Leasing Adoption
Cheng (2021) [34]	Residential land lease	Valuation strategies
Hoogland et al. (2022) [35]	Hybrid electric vehicles	Leasing decision making
Ayinaddis (2022) [28]	Electric vehicles	Environmental impacts of leasing
Palm-Forster et al. (2023) [36]	Cropland Leasing	Optimization of lease contracts
Cote et al. (2023) [37]	Heat pumps	Leasing intention
Lu et al. (2023) [38]	Chemical leasing	Leasing adoption
Liu et al. (2023) [39]	Second-life batteries	Recycling efficiency optimization
Onofri et al. (2023) [40]	Agricultural land leasing	Optimization of lease contracts
Deininger et al. (2023) [41]	Public land lease	Public Policy
Gonzalez-Salazar et al. (2023) [42]	Electric vehicle leasing	Environmental impacts of leasing

### 2.3 Extension of UTAUT2

With the rapid development of information and communication technology and artificial intelligence technology, various applications based on artificial intelligence have become an indispensable part of the operation of small and medium-sized enterprises. Understanding the acceptance of computing power leasing by SMEs should be the latest research direction for the development of SMEs. At present, there are many theories that explain the technology adoption behavior of SMEs, such as the Theory of Planning Behavior (TPB), the Technology Acceptance Model (TAM), and Unified Theory of Acceptance and Use Technology (UTAUT). Venkatesh et al. proposed and tested Unified Theory of Acceptance and Use Technology 2 (UTAUT2), adding new elements to the UTAUT model, namely hedonic motivation (HM), price value (PV), and habit (HB). The prediction ability of UTAUT2 theory is much higher than that of UTAUT theory. Table 3 shows the research applications of UTAUT2 in different fields, which has a strong reference value for the establishment of the research model.

**Table 3:** Previous researches of extending UTAUT2.

Authors	Extending factors	Research Object
Rahi, S. , & Abd. Ghani, M. (2018) [43]	Innovativeness, technology security	Internet banking
Kapser & Abdelrahman (2020) [44]	Perceived risk, Price sensitivity	Last mile delivery
Yang et al. (2022) [45]	Attitude	Basketball learning
Schmitz et al. (2022) [46]	Perceived security, Product advantage	Telemedicine adoption
Korkmaz et al. (2022) [47]	Trust & safety, Perceived Risk	Public transport system adoption
Medeiros et al. (2022) [48]	Privacy, Help, Benefits, Rewarding, Self-image	Mobile application usage
Arpaci et al. (2022) [49]	Extraversion, openness, Neuroticism	Social sustainability
Singh et al. (2023) [50]	Personal norm, Awareness	Electric vehicle adoption
Martinez & Mcandrews, (2023) [51]	Risk	Mobile pay adoption
Sebastian et al. (2023) [52]	Risk, Trust, Security	Mobile pay adoption
Barua & Barua (2023) [53]	Health, Constraint	Mobile health adoption
Kilani et al. (2023) [54]	Trust	E-wallet adoption
Sun et al. (2023) [55]	Knowledge, risk, Innovativeness	Delivery application adoption
Shareef et al. (2024) [56]	Effort value	Mandatory technology adoption

### 2.4 Task-technology fit theory

Task-technology fit (TTF) theory is a method that describes whether information technology tools can effectively support specific tasks based on the concept of task technology and adaptation. It involves four key elements: task characteristics, technology characteristics, user characteristics, and task technical adaptation. Together, these elements influence the final behavior or use. TTF model is to determine whether the functionality of information technology is effective in supporting the behavioral needs of users. Information technology can only be adopted and used if its functions meet the task needs of the user. In addition, the TTF model can also be used to evaluate and predict the utilization efficiency of network information resources, taking into account the matching relationship between information system functions and user task requirements. Table 4 points out that many scholars have extended this model, such as the impact of user self-efficacy on adaptation, the influence of organizational environment on adaptation, and the integration with other models.

**Table 4:** Previous researches about TTF.

Authors	Research factors	Research Object
Liu et al. (2011) [57]	Attitude, Performance, Task-individual fit	Decision support system
Parkes (2013) [58]	Attitude, Performance, Decision quality	Decision support performance
Khan et al. (2018) [59]	Reputation, Relatedness, Autonomy	Online education adoption
Li et al. (2019) [60]	Dependency, Communication, Play	Mobile Social media usage
Rai & Selnes (2019) [61]	Ease of use, Usefulness, Adopt, Norm	Digital text book adoption
Howard & Rose (2019) [62]	Reactions, Utilization, Performance outcome	Theory refinement
Erskine et al. (2019) [63]	Ability, Motivation, Self-efficacy, Advantage	Decision making performance
Rahi et al. (2020) [64]	Satisfaction, User expectation	Internet banking
Wang et al. (2021) [65]	Pre and post-shopping Trust	Shopping behavior
Jeyaraj (2022) [66]	Type of respondents, Type, of technology	Information system research
Muchenje& Seppanen (2023) [67]	Stable interaction, Task, Interaction	Big data analytics
Shahzad et al. (2023) [68]	Self-efficacy, Rating, Attitude, Design	Mobile food delivery service
Ruyobeza et al. (2023) [69]	Competence, Skills, Self-efficacy	Digital health technologies
Koh et al. (2023) [70]	Privacy, Risks, Attitude, Usefulness	Urban drone adoption
Alyoussef (2023) [71]	Enjoyment, Usefulness, Quality, Ease of use	E-learning in higher education
Ayyoub et al. (2023) [72]	Usefulness, Satisfaction, Confirmation	Online assessment
Cheikh-Ammar (2024) [73]	IT spirit, IT affordance, Task requirement	User evaluation

Based on the above discussion, it can be concluded that although the two theories of UTAUT2 and TTF are often used in adoption researches, they are often extended or combined with other theories to better explain adoption behavior. This study combines and extends the two theories so as to better explore the high-tech SMEs Adoption of Artificial Intelligence Computing Power Leasing Business.

## 3 Hypothesis development

### 3.1 SME innovativeness and usage

SME innovativeness means SMEs are comfortable with high unfamiliarity but will take the risks [54]. In the face of the surge in computing power demand brought about by the wide application of artificial intelligence technology, especially large models, many small and medium-sized enterprises are feeling great pressure. Intelligent computing power has become the core force driving AI large model training, and the imbalance between supply and demand of high-performance graphics processing units is the key to meeting this demand, which has led to rising prices and longer lead times. This situation has caused many small and medium-sized enterprises to encounter bottlenecks on the road to the pursuit of computing power.

In the face of this challenge, SMEs must be open-minded and adopt innovative behaviors to compensate for this disadvantage. The emergence of the computing power leasing model provides a new solution for enterprises. Thanks to the increasing maturity and popularity of cloud computing technology, computing power leasing has become an ideal choice to provide users with powerful computing power in a flexible and on-demand manner. It allows users to access the computing power they need anytime, anywhere through the cloud, which not only greatly enhances the scalability and flexibility of computing power, but also realizes true pay-as-you-go, saving a lot of costs for enterprises.

When small and medium-sized enterprises face severe market competition and performance completion pressure, they are likely to innovatively adopt AI computing power leasing to meet production needs. Innovation is an important factor for enterprises to stand out from the competition, and many previous studies have taken innovation as the core of enterprise development [15,21,22,25,27,28,30]. Therefore:

H1 SME innovativeness positively affects the usage of SMEs' AI computing power leasing.

### 3.2 Perceived risk and usage

Perceived risk of SMEs is the likelihood that they may suffer loss from AI computing power leasing [74]. Perceived risk is often used to extend UTAUT2 theory [44,46,47,51,52,75] and is considered an important factor in the adoption of behavioral research. For those SMEs with computing power needs, renting cloud computing power can not only significantly reduce hardware costs, but also improve the ability to control costs. In addition, computing power migration to the cloud also lays the foundation for efficient computing power scheduling. For enterprises focusing on the research and development of small models in the vertical industry, the computing power leasing model is a powerful tool to lower the threshold for the research and development of large models.

In spite of these advantages, the SMEs must know that the lessor's main profit source is the difference between rental income and operating costs. The operating costs of the lessor cover a variety of aspects, including the depreciation expense of fixed assets, the daily operating expenses of the data center, the rent of the computer room, and the cost of personnel. Since AI graphics cards are replaced quickly, lessors must recoup their AI graphics cards before they become obsolete. Based on this pricing idea, the lessor will evaluate the payback period of the overall investment in computing power leasing, and may adjust the price or service method according to the supply and demand relationship. All the core data of small and medium-sized enterprises will be uploaded to the lessor, which brings data security problems. The User may wonder whether the Lessor has sufficient capacity to ensure that it does not infringe the User's intellectual property rights. All of the above uncertainties may increase the user's perceived risk, thereby reducing the user's enthusiasm for computing power leasing. Thus:

H2 Perceived risk negatively affects the usage of SMEs' AI computing power leasing.

### 3.3 Performance expectation and usage

Performance expectation is the degree that SMEs perceive when leasing AI computing power [44]. Globally, the U.S. has imposed a technology embargo and blockade on China, leaving China's AI development and application industry to make more use of leasing. In particular, there is a big performance gap between the graphics processing units produced in China and the advanced graphics processing units abroad, and they cannot be mass-produced in the short term, so the current development of China's AI industry still needs to rely mainly on advanced graphics processing units represented by overseas NVIDIA Corporation and AMD Corporation. In the process of AI computing power leasing, users can choose the type and time of resources to use according to their needs, and do not need to bear the corresponding costs such as operation, maintenance, and upgrades. On the one hand, AI computing power leasing lowers the threshold for users to use it, which is more conducive to the rapid development and application of products and services by many SMEs, and on the other hand, it also makes full use of idle computing resources to improve the utilization efficiency of computing resources. There is no doubt that leasing not only improves the performance expectation of using AI for SMEs, but also improves the utilization rate of equipment for lessors. Therefore:

H3 Performance expectancy positively affects the usage of SMEs' AI computing power leasing.

### 3.4 Price value and usage

Price value is the SMEs trade-off between their perceived benefits and cost in using AI computing power leasing [75]. The emergence of the AI computing power leasing model provides a new solution for SMEs. Thanks to the increasing maturity and popularity of cloud computing technology, AI computing power leasing has become an ideal choice to provide users with powerful computing power in a flexible and on-demand manner. It allows users to access the computing power they need anytime, anywhere through the cloud, which not only greatly enhances the scalability and flexibility of AI computing power, but also realizes true pay-as-you-go, saving a lot of costs for SMEs. One of the outstanding features of China's economy is that the government plays an important role in the operation of the market economy. In some technical fields, the government's investment and allocation of technological resources directly affect the efficiency and effectiveness of the operation of the market economy. Executives of small and medium-sized high-tech enterprises may also consider the visa value brought by the policy while adopting the degree of adaptability of computing power leasing to their own tasks. For SMEs that focus on the research and development of small models in the vertical industry, the computing power leasing model is a powerful tool to lower the threshold for the research and development of large models. With its flexibility, scalability and cost-effectiveness, the computing power leasing model has become the best choice for SME computing power supply in the face of sanctions. Thus:

H4 Price value positively affects the usage of SMEs' AI computing power leasing.

### 3.5 Task-technology fit and usage

The earliest TTF model was only aimed at the problem of adaptation between an individual's use of technology and the demands of his task. Through the cognitive psychology and cognitive behavior of executives of small and medium-sized high-tech enterprises, the logical relationship between computing technology support for their daily research and development tasks and performance improvement is revealed, and the TTF theory is introduced into the core of this study. However, in the process of completing many specific tasks, users are more likely to use a member of the group or organization as the user of the technology. More and more researches on technology adoption behavior integrate TTF with other theories and are widely used in various research fields. Khan et al. (2018) [59] combine TTF model with self-determination theory to find factors affecting e-learning adoption. Rai & Selnes (2019) [61] integrate TTF model with technology acceptance model to test adoption of digital textbook service. Alyoussef (2023) [71] extends TTF model with information system success model to test the acceptance of e-learning. Koh et al. (2023) [70] uses TTF model with pricacy calculus theory to find factors affecting urban drone adoption. Cheikh-Ammar (2024) [73] develops a new theory of IT desirability based on TTF to explain person-IT fit. Since computing power leasing can be used on demand, it can greatly reduce the cost of use, especially for small and medium-sized enterprises or individual users. The lessor will provide round-the-clock customer support services, including online consultation, telephone support, remote support and other forms, to respond to users' questions and feedback in a timely manner, and assist users in resolving faults. In particular, for SMEs with limited budgets [76], it is crucial to design an efficient and low-cost leasing solution to solve daily tasks, as the volume of their tasks does not remain the same, but changes according to the order. Obviously, when SMEs can design technical requirements based on their own task characteristics, they will better use AI computing power leasing. Therefore:

H5 Task-technology fit positively affects the usage of SMEs' AI computing power leasing.

### 3.6 Moderating effects of task-fit technology fit

Many previous researches have extended TTF model with moderating factors to better explain adoption behaviors. Khan et al. (2018) [59] use reputation as a moderating factor to extend TTF model, whereas Howard & Rose (2019) [62] test utilization's moderating effect in a refined TTF model. Wang et al. (2021) [65] make technology as a moderator in a shopping technology adoption research; meanwhile, self-efficacy is applied to TTF model as a moderator in delivery research by Shahzad et al. (2023) [68]. When SMEs have a better task-technology fit, they may have more manpower, material resources, and energy to carry out technological innovation in order to make better use of AI computing power leasing. This progress indicates that if task-technology is high, the SME innovativeness may more positively affect usage. Thus:

H6 Task-technology fit positively moderates the relationship between SME innovativeness and usage of AI computing power leasing.

A better task-technology fit means that SMEs and the lessor have a good working relationship, and both parties can maintain the efficiency of technical support for the task in a timely manner with the usual communication mechanism, and flexibly adjusts the cooperation mechanism between the two parties. This efficient technology service task mechanism can greatly improve the cooperation between the two parties and reduce the probability of error, thereby greatly reducing the risk perception of SMEs and improving the efficiency of use. Hence, when task-technology fit is high, the degree of impact of perceived risk on use could be significantly reduced. Therefore:

H7 Task-technology fit negatively moderates the relationship between SME perceived risk and usage of AI computing power leasing.

AI computing power leasing not only makes personalized services more popular, such as users can change the service content at any time according to their needs to better improve work performance, but also can switch between different rental service providers. When computing power leasing can serve the work well, more engineers can be liberated from the work of building AI computing power platforms and concentrate on design and development, thereby greatly improving work performance. Thus, when task-technology fit gets higher, performance expectancy could affect usage more. Therefore:

H8 Task-technology fit positively affects the relationship between performance expectancy and usage of AI computing power leasing.

Due to the shortage of supplies caused by sanctions, the price of AI computing graphics cards has skyrocketed in China, and SMEs simply cannot afford the high cost of building AI hardware platforms. The emergence of AI computing power leasing is not only one of the best choices for SMEs, but also the only support for Chinese SMEs to catch up with the world's AI application development. The competition in the Chinese market is fierce due to the fact that many government-backed enterprises and financially strong listed companies carry out AI computing power leasing business. This competition is good news for SMEs because it gives them more options to find services that are more suitable for their type of task at a reasonable price. This shows that when task-technology gets higher, the price value will have a more positive impact on the use. Thus:

H9 Task-technology fit positively affects the relationship between price value and usage of AI computing power leasing.

**Fig. 1:**
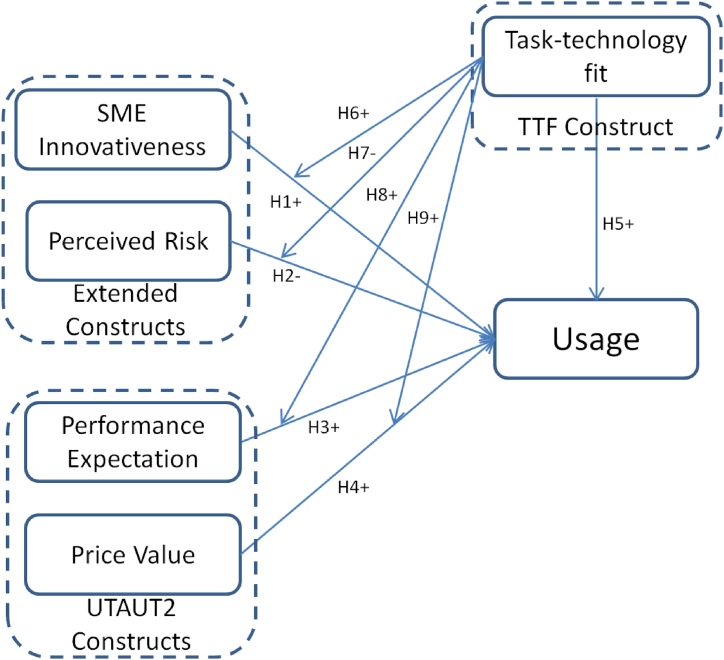
Research model.

Fig. 1 shows the research model. In this model, task-technology fit is a construct from task-technology fit theory; whereas performance expectancy and price value factors come from UTAUT2 theory. SEM innovativeness and perceived risk are two extended constructs to better explain the adoption behavior of SMEs’ AI computing power leasing because a multi-theory research model is always applied to profound adoption behavior research. Wang et al. (2021) [77] combine TTF theory with push-pull-mooring theory to test factors affecting shopping behaviors for healthcare. Faqih & Jaradat (2021) [78] also integrate TTF theory and UTAUT2 theory to find factors that may affect augmented reality technology acceptance.

## 4 Methods

### 4.1 Research design and data collection

This survey is a questionnaire for small and medium-sized high technology companies in Chinese mainland engaged in AI industry development. This study is purely commercial research and does not contain any information related to animal, plant and human health. All the content and format of the investigation in this study are not only fully compliant with legal procedures and ethical constraints, but the relevant guidelines and regulations are well enforced. All participating companies have given permission and consent to the use of the survey data for the purposes of this study, including the questionnaire and any related details regarding various ethical behaviors.

The companies that participated in the questionnaire were distributed in different provinces of middle and east China including Henan, Jiangsu, Shandong and Anhui. These small and medium-sized high-tech enterprises include: aviation parts manufacturing, ship parts manufacturing, machine tool manufacturing, industrial crystal production, circuit board production, landscape design and 6G communication companies etc.

In this study, a multivariate sampling technique was adopted, and samples from different provinces and industries were selected. That is, samples with different characteristics are selected to better represent the population. The sample size of this study is relatively large, because increasing the sample size can reduce the sampling error and improve the accuracy and reliability of the results.

Because the companies participated anonymously, the researchers were unable to obtain any private information about the participants. The questionnaire is distributed to the CEOs of the enterprises through the Internet, and they enter the questionnaire filling system by scanning the QR code. Each CEO who participates in the filling will receive a movie voucher as a thank you. The questionnaire starts on November 29, 2023 and ends on January 5, 2024. A total of 387 questionnaires were received, of which incomplete questionnaires and invalid questionnaires were issued, leaving 281 valid questionnaires, which mean that the questionnaires filled in by the CEOs of 281 companies were used for subsequent data analysis.

### 4.2 Measurement

In order to improve the reliability and validity of the studies, one of the common measures is to design measurement items based on previous studies. SME innovativeness has three items based on previous research of Sun et al. (2023) [55]: 1. Our company is always the first to try it out among other colleagues and peers; 2. Overall, our company would like to try and experiment with new things; 3. The belief in being ahead of our peers drives our company to look ways to experiment with AI power computing leasing service. Perceived risk has three items based on previous research of Wu et al. (2022) [79]: 1. AI computing power lessors misuse our data is risky for me; 2. It is not wise for AI computing power lessors to collude to raise prices; 3. It costs a lot to find high quality and affordable AI computing power leasing services. Performance expectation has three items based on previous research of Venkatesh et al. (2016) [80]: 1. Our company finds AI computing power leasing service useful in daily work; 2. Using AI computing power leasing service would help our company accomplish things more quickly; 3. Using AI computing power leasing service would increase our company's productivity and flexibility in daily work. Price value has three items based on Faqih & Jaradat (2021) [78]: 1. Using AI computing power leasing service in daily work would be available at reasonable price; 2. Using AI computing power leasing service in daily work would provide our company with good value for money; 3. Using AI computing power leasing service in daily work provides a good value at a current price. Task-technology fit has three items based on Wang et al. (2021) [77]: 1. AI computing power leasing services are sufficient in helping our company to complete daily work; 2. AI computing power leasing services are appropriate in helping our company to complete daily work; 3. In general, AI computing power leasing services could fully meet our daily work. Usage has three items based on Sun et al. (2020) [81]: 1. I am likely to use AI computing power leasing services; 2. I desire to use AI computing power leasing services; 3. I plan to use AI computing power leasing services.

This research applies Smart-PLS 4 to data analysis progress because it has several advantages: 1. It is widely used in various scientific research fields: marketing, behavioral finance, marketing, information systems, organizational behavior and other fields; 2. It is often used for new research subjects and is good at small-scale data analysis; 3. It has a relatively excellent human-computer interface and has many ready-made research models to choose from; 4. It has relatively excellent calculation speed and low requirements for data preprocessing. Based on these advantages, as well as the fact that AI computing power rental service is a relatively new research object, and considering the paradigms and sampling commonly used in the field of business research [82], it is used as a research tool.

## 5 Results

Table 5 gives the demographic statistics of the survey. Most of the CEOs of high-tech SMEs are males; whereas there are about 78.2 % of them have a master or PH.D degree. Because AI computing power leasing started after China was hit by trade sanctions in recent years, most SMEs lease AI computing power for 6 months to 2 years. Although these SMEs are all engaged in AI application development, their application focus is different. More than half of the computing power leased by SMEs is used for deep learning and scientific computing. More than half of the AI computing power leased by enterprises is used for deep learning and scientific computing, and 17.8 % of enterprises are engaged in 6G software development to meet the needs of AI mobile phone applications. The diversity of uses reflects the high popularity of AI computing power leasing in more industries.

**Table 5:** Demographic statistics.

Category	Subject	N	%
Gender	Male	209	74.4 %
	Female	72	25.6 %
Education level	High school	0	0 %
	Bachelor	61	21.8 %
	Master	198	70.4 %
	Ph.D.	22	7.8 %
Age	20–30	8	2.8 %
	31–40	91	32.4 %
	41–50	148	52.6 %
	More than 50	34	12.2 %
Term of leasing AI computing power	<6 months	78	27.7 %
	6–12months	111	39.5 %
	12-24moths	69	24.5 %
	>24 months	23	8.3 %
Usage of leasing AI computing power	Deep learning	65	23.1 %
	Scientific computing	81	28.8 %
	Video processing	47	16.7 %
	Graphic visualization	31	11.0 %
	6G software development	50	17.8 %
	Others	7	2.6 %

Table 6 examines the reliability and validity of the questionnaire data. Average Variance Extracting (AVE) is a validity test that indicates the validity of the study data when all values are greater than 0.5. Composite Reliability and Cronbach's α are commonly used to measure the reliability of a research model, and when their values are above 0.7, the research model as a whole is considered reliable. The data in Table 6 all meet these conditions, so the reliability of the studied model is passed.

**Table 6:** Convergent validity, composite reliabilities testing results (INN-SME Innovativeness, PR-Perceive Risk, PE-Performance expectation, PV-Price value, TTF-Task technology fit, U-usage).

Construct	Item	Standard deviation	AVE	Composite Reliability	Cronbach's α
SME Innovativeness	INN1	0.003	0.927	0.975	0.961
	INN2	0.003			
	INN3	0.004			
Perceived Risk	PR1	0.153	0.916	0.970	0.963
	PR2	0.173			
	PR3	0.155			
Performance Expectation	PE1	0.002	0.963	0.987	0.981
	PE2	0.003			
	PE3	0.003			
Price Value	PV1	0.002	0.967	0.984	0.983
	PV2	0.003			
	PV3	0.002			
Task technology fit	TTF1	0.004	0.968	0.985	0.983
	TTF2	0.004			
	TTF3	0.003			
Usage	U1	0.009	0.952	0.978	0.975
	U2	0.012			
	U3	0.006			

Table 7, Table 8, Table 9 are used to test the Discriminant validity of research model. Discriminant validity means that when different methods are applied to measure different variables, the observed values should be able to distinguish between them. Table 7, Table 8, Table 9 are used together to demonstrate the process of discriminant validity testing. When the following three conditions are met at the same time, the discriminant validity test of the research model can be passed: 1. all values in Table 7 are less than the threshold of 0.85; 2. all absolute values in Table 8 are less than the threshold of 1.0, 3. All values on the left in Table 9 are less than the diagonal value in the same row. Fortunately, the above conditions are met in Table 7, Table 8, Table 9, so the discriminative validity of the study model is tested.

**Table 7:** Discriminant validity (Heterotrait–Monotrait ratio).

	PV	PR	PE	INN	TTF	U	TTF *INN	TTF *PR	TTF *PV	TTF *PE
PV										
PR	0.128									
PE	0.534	0.269								
INN	0.558	0.027	0.446							
TTF	0.435	0.178	0.308	0.353						
U	0.408	0.055	0.338	0.343	0.274					
TTF*INN	0.163	0.019	0.092	0.470	0.249	0.165				
TTF*PR	0.105	0.202	0.169	0.010	0.246	0.153	0.123			
TTF*PV	0.351	0.104	0.219	0.189	0.381	0.140	0.439	0.177		
TTF*PE	0.198	0.159	0.337	0.096	0.345	0.126	0.296	0.366	0.574

**Table 8:** Bootstrapping confidence interval up of HTMT.

	2.5 %	97.5 %
PV-U	0.099	0.385
PR-U	−0.271	−0.006
PE-U	0.052	0.347
INN-U	0.037	0.346
TTF-U	0.087	0.350
(TTF*INN)-U	0.021	0.279
(TTF*PR)-U	−0.229	−0.031
(TTF*PV)-U	0.014	0.311
(TTF*PE)-U	0.011	0.279

**Table 9:** Discriminant validity-Fornell Larker criterion.

	PV	PR	PE	INN	TTF	U
PV	0.983					
PR	0.107	0.957				
PE	0.524	0.246	0.981			
INN	0.543	−0.026	0.433	0.963		
TTF	0.428	0.162	0.303	0.346	0.984	
U	0.401	−0.075	0.332	0.335	0.268	0.975

Table 10 gives the Mean, Standard deviation and P values of the results, which indicates that all the P values are less than 0.05. This means that all the hypotheses are supported. Multi-collinearity is tested by Variance Inflation Factor (VIF). When values if VIF are all less than 5, it is indicated that there is no collinearity problem in the research model.

**Table 10:** Mean, standard deviation and P values (INN-SME Innovativeness, PR-Perceive Risk, PE-Performance expectation, PV-Price value, TTF-Task technology fit, U-usage).

	Sample mean	Standard deviation	P values	VIF
PR-U	−0.145	0.070	0.024	1.131
PE-U	0.190	0.077	0.012	2.166
PV-U	0.237	0.074	0.001	2.665
INN-U	0.194	0.079	0.017	2.124
TTF-U	0.212	0.068	0.002	1.461
TTF*INN-U	0.139	0.065	0.023	1.827
TTF*PR-U	−0.110	0.051	0.023	1.307
TTF*PE-U	0.139	0.068	0.038	2.538
TTF*PV-U	0.169	0.076	0.033	2.727

**Fig. 2:**
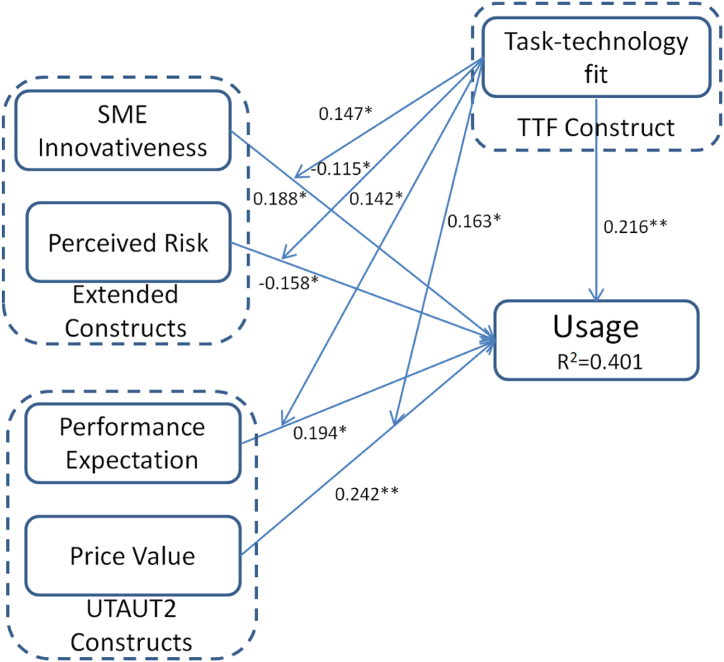
Empirical test results of research * (P < 0.05) ** (P < 0.01).

Fig. 2 is the description of empirical test results of research. Path coefficient value SME innovativeness is positively related to usage with a path coefficient value 0.188. Perceived risk is negatively related to usage with a path coefficient value −0.158. The P values of the two relation significance are both less than 0.05. These results indicate that extended constructs are effective supplement to TTF and UTAUT2 theories in this research model. Interestingly, both the performance expectation and price value positively related to usage with path coefficient values 0.194 and 0.242. Price value has a more significant value (P < 0.01) than performance expectation (P < 0.05). Task technology fit not only positively related to usage but also has significant moderating effects between other constructs and usage. The result indicates that the path coefficient between task technology fit and usage is 0.216 with P value less than 0.01. Task technology fit positively moderates the relationship between SME innovativeness and usage with path coefficient value 0.147 and P value less than 0.05. Task technology fit negatively moderates the relationship between perceived risk and usage with path coefficient value −0.115 and P value less than 0.05. Task technology fit positively moderates the relationship between performance expectation and usage with path coefficient value 0.142 and P value less than 0.05. Task technology fit positively moderates the relationship between SME innovativeness and usage with path coefficient value 0.163 and P value less than 0.05. The adjusted R-squared value is the degree to which the variables in the overall model as a whole can explain usage. The results show that the variables of the overall model can explain 40.1 % of the usage.

## 6 Conclusion

### 6.1 Comprehensive discussion

SME innovativeness is positively related to usage, which indicates that businesses that dare to try new things and have an adventurous spirit are more willing to use AI computing power leasing services. In order to increase the motivation of SMEs to actively innovate, computing power leasing companies could subdivide the rental equipment, such as leasing the entire server, leasing by the scale of computing power, or paying by the number of graphics processing units rented. This kind of segmentation service allows SMEs to purchase the most cost-effective services at any time according to their own needs, thereby greatly improving SME's independent innovation capabilities. Furthermore, Computing power leasing companies can assist in the design of AI software according to the needs of SMEs, such as SaaS (Software as a Service), which allows SMEs to use cloud-based design-assisted functions through an Internet connection.

Many across border companies that engaged in AI computing power leasing business are interested in the low threshold of this business [83]. However, from the perspective of customers, there may be certain risks in the computing power leasing services provided by such cross-border companies. The above points also correspond to the results of the study, which is that perceived risk is negatively related to usage. Due to sanctions, most of the companies in China that have entered the computing power leasing industry cannot get a stable supply of AI graphic cards. When the computing power chip equipment of these enterprises is facing aging or upgrading, SMEs may face the risk of having no computing power to rent. Many AI leasing companies have recognized this shortcoming, and they either strengthen their ties with foreign chip sales, or invest a lot of manpower and material resources in the research and development of AI chips and servers, such as Huawei's Ascend series chips, so as to fundamentally eliminate the hidden danger of computing power shortage. The government or industry organizations should formulate policies to protect the information of SMEs from being infringed, because a large number of SMEs' business information may be transmitted to the AI graphics card for processing.

It is easy to understand that performance expectancy is positively related with usage, as AI computing power leasing is currently an effective way for Chinese SMEs to obtain sufficient AI computing power to carry out their work. Due to the recent purchase of AI graphics cards by many companies to enter the field of computing power leasing [84], the lessor market is facing considerable competitive pressure. In order to provide customers with better services that enhance their performance expectations, the lessor can customize computing power services according to users’ needs, such as ordering different types of computing graphics cards or conducting AI programming outsourcing services according to customer requirements.

Although the positive relationship between price value and usage suggests that users are accepting of the current diverse AI computing power leasing models, the potential threat is that the prices of the latest AI computing chips are constantly rising. The sharp rise in chip prices leading to fluctuations in rental prices can lead to instability in the entire AI computing power rental market. This requires the Chinese government to introduce a series of subsidy policies for users and lessors in the computing power leasing market to stabilize leasing prices and promote the healthy development of the entire market. A better vision for the future is that AI computing chips in China can be supplied to the market as quickly and steadily as possible at relatively low prices, and this situation is also based on strong government support.

The empirical results show that task technology fit is not only positively correlated with usage, but also has a significant moderating effect on the other four relationships. While AI computing leasing proves to be a great fit for the daily work of high-tech SMEs, there are many more details that the lessor can improve. The competitive barriers of American companies' AI chips are not only reflected in the chip manufacturing process and system integration, but more importantly, the long-term accumulation in the software ecosystem. Many Chinese algorithm engineers have become accustomed to the tool libraries and programming languages provided by American companies, and it is difficult for them to accept new programming languages. Even though China's domestic AI chips are gradually replacing imported chips, the development platform and programming language should be consistent with previous applications. Lessors can design more product leasing and payment models, as multiple options allow lessees to find products that are more suitable for their work tasks and greatly reduce the risk of cost waste, thus greatly improving the ability to innovate work. Partnering with telecommunications companies to customize private network lines for those companies with a high focus on information security is an effective way to address information security anxiety. Although the price of the latest AI computing graphics card is rising, the lessor can price the product according to different time periods. For companies that do not need immediate results, the lessor can perform their calculations at night or during other free time. This method of pricing according to the characteristics of the customer's task can greatly improve the price competitiveness of the product.

### 6.2 Theoretical implications

TTF theory is often combined with other theories to explain new applications of adoption research, such as E-Learning adoption [85]. However, the combination of TTF and UTAUT theories to explain the latest adoption of computing power leasing is still rare. TTF The theoretical contribution of the combination of UTAUT and TTF are also reflected in the exploration and development of the development path of high-tech enterprises with Chinese characteristics. China's counter-sanctions strategy is a unique development path explored in accordance with China's national conditions and the characteristics of the times. The combination of UTAUT and TTF not only explains the core factors of China's current sanctions resolution, but also combines some of the current characteristics of China's small and medium-sized enterprises to form a high-tech development system with Chinese characteristics, which provides a strong theoretical guarantee for China to resolve economic sanctions in the future.

### 6.3 Practical implications

The proposal and implementation of computing power leasing is a major strategic contribution of historical significance made by China to participate in and improve the global economic governance system in the context of the in-depth development of global economic integration. As a new cross-industry cooperation model, computing power leasing is of great significance in both theory and practice for the competition and development of China's high-tech SMEs in both domestic and foreign markets.

After the reform and opening up, China has made remarkable achievements in economic and social development, and in the process, it has gradually integrated into the world trade, financial and investment system, and China has become an economy with a decisive position in the world. In recent years, with the establishment of its status as the world's second largest economy, China has developed into an “economic growth pole” that has contributed the most to world economic growth, and has an increasing voice in global economic governance, and has a leading position in the direction of the world economy. However, with the United States' economic sanctions against China, China's high-tech industry has encountered difficulties in its development, especially the AI industry, which has encountered a big computing power bottleneck. The smooth advancement and steady implementation of the computing power leasing industry has provided a new solution for China's economic recovery in the predicament and the construction of China's dual identity as a “world factory” and a “world market”, which is a great theoretical contribution.

## 7 Limitations and future research

Although this research makes some progresses in AI computing leasing adoption, there are still some limitations. Firstly, this study focuses only on SMEs and neglects powerful state-owned enterprises. State-owned enterprises may not adopt a leasing model, but build their own computing centers. In the future, the preference of state-owned enterprises in terms of computing power can be studied. Secondly, this study is based on AI chips produced in the United States, and in the future, researches can be carried out on the SME adoption of AI chips produced in China. Thirdly, the sanctions are aimed at companies in China, and if SMEs move out of China to develop AI programs in neighboring countries, then all sanctions will be invalidated. Future research can focus on the feasibility of SME setting up computing centers in China's neighboring countries.

## Funding/acknowledgement/conflict of interest information

1. There is no funding in this research.
2. No conflict of interest exits in the submission of this manuscript, and manuscript is approved by all authors for publication.

## Data availability statement

Data is included in Supplementary Materials.

## CRediT authorship contribution statement

**Wei Sun:** Writing – review & editing, Writing – original draft, Funding acquisition, Formal analysis, Data curation, Conceptualization. **Alisher Tohirovich Dedahanov:** Project administration, Methodology, Investigation, Funding acquisition. **Wei Ping Li:** Validation, Supervision, Software, Resources, Project administration. **Ho Young Shin:** Writing – review & editing, Writing – original draft, Visualization, Validation.

## Declaration of competing interest

The authors declare the following financial interests/personal relationships which may be considered as potential competing interests:Wei Sun reports article publishing charges was provided by Henan University of Urban Construction. If there are other authors, they declare that they have no known competing financial interests or personal relationships that could have appeared to influence the work reported in this paper.
